# The COVID-19 Infection Diffusion in the US and Japan: A Graph-Theoretical Approach

**DOI:** 10.3390/biology11010125

**Published:** 2022-01-13

**Authors:** Mohammad Reza Davahli, Waldemar Karwowski, Krzysztof Fiok, Atsuo Murata, Nabin Sapkota, Farzad V. Farahani, Awad Al-Juaid, Tadeusz Marek, Redha Taiar

**Affiliations:** 1 Department of Industrial Engineering and Management Systems, University of Central Florida, Orlando, FL 32816, USA; wkar@ucf.edu (W.K.); fiok@ucf.edu (K.F.); 2 Department of Intelligent Mechanical Systems, Graduate School of Natural Science and Technology, Okayama University, Okayama 700-8530, Japan; a.murata.koma@gmail.com; 3 Department of Engineering Technology, Northwestern State University of Louisiana, Natchitoches, LA 71459, USA; sapkotan@nsula.edu; 4 Department of Biostatistics, Johns Hopkins University, Baltimore, MD 21218, USA; ffaraha2@jhu.edu; 5 Industrial Engineering Department, Taif University, Taif 26571, Saudi Arabia; amjuaid@tu.edu.sa; 6 Department of Cognitive Neuroscience and Neuroergonomics, Institute of Applied Psychology, Jagiellonian University, 30-348 Kraków, Poland; marek@uj.edu.pl; 7 Matériaux et Ingénierie Mécanique (MATIM), Université de Reims Champagne-Ardenne, 51100 Reims, France; redha.taiar@univ-reims.fr

**Keywords:** COVID-19 pandemic, graph theory, pandemic diffusion, network density

## Abstract

**Simple Summary:**

In this study, we conducted a quantitative assessment and compared the COVID-19 pandemic spread in two countries based on selected methods from the graph theory domain. The results indicate that while the applied experimental procedures are useful, we could draw limited conclusions about the dynamic nature of infection diffusion. We discussed the possible reasons for the above and used them to formulate research hypotheses that could serve the scientific community in future research efforts.

**Abstract:**

Coronavirus disease 2019 (COVID-19) was first discovered in China; within several months, it spread worldwide and became a pandemic. Although the virus has spread throughout the globe, its effects have differed. The pandemic diffusion network dynamics (PDND) approach was proposed to better understand the spreading behavior of COVID-19 in the US and Japan. We used daily confirmed cases of COVID-19 from 5 January 2020 to 31 July 2021, for all states (prefectures) of the US and Japan. By applying the pandemic diffusion network dynamics (PDND) approach to COVID-19 time series data, we developed diffusion graphs for the US and Japan. In these graphs, nodes represent states and prefectures (regions), and edges represent connections between regions based on the synchrony of COVID-19 time series data. To compare the pandemic spreading dynamics in the US and Japan, we used graph theory metrics, which targeted the characterization of COVID-19 bedhavior that could not be explained through linear methods. These metrics included path length, global and local efficiency, clustering coefficient, assortativity, modularity, network density, and degree centrality. Application of the proposed approach resulted in the discovery of mostly minor differences between analyzed countries. In light of these findings, we focused on analyzing the reasons and defining research hypotheses that, upon addressing, could shed more light on the complex phenomena of COVID-19 virus spread and the proposed PDND methodology.

## 1. Introduction 

China officially reported the first case of a new coronavirus disease, COVID-19, on 8 December 2019 [1]. As China failed to control the outbreak, the virus responsible for this disease, SARS-CoV-2, spread to many countries and was declared a global pandemic [2,3]. The US and Japan are among the countries affected by the SARS-CoV-2 virus. 

The US reported its first confirmed case of COVID-19 on 20 January 2020 [4]. By the end of January, the number of confirmed cases increased to six; consequently, the US government restricted travel from China and declared a public health emergency [5]. By the end of February, the number of confirmed COVID-19 cases had grown to 60; on 13 March, the case number climbed above 2100, and the US administration declared a national emergency due to the COVID-19 outbreak [5]. 

From January 2020 to July 2021, the US faced three waves of SARS-CoV-2 infections. The first wave was from 20 March to 10 June 2020, with 2,104,956 confirmed cases and 118,464 deaths; the second wave was from 10 June to 16 September 2020, with 4,889,694 confirmed cases and 84,521 deaths; and the third wave was from 16 September 2020 to 20 June 2021, with 27,300,183 confirmed cases and 414,833 deaths [6]. 

To address the pandemic, the US federal and state governments focused on four key measures: (1) investing in research to accelerate the production of vaccines, diagnostics, and treatments; (2) improving access to diagnostics and treatment; (3) improving health system delivery to rapidly respond to the COVID-19 outbreak; and (4) increasing the availability of data to improve surveillance [7]. However, the US was not successful in implementing all key measures, which have been only partially addressed. The US was successful in mainly increasing funding for scientific research, developing vaccines, and changing regulations regarding telemedicine; however, the US is falling behind in developing unified policies among all states [7]. 

Japan reported its first confirmed case of SARS-CoV-2 infection on 16 January 2020 [8]. By the end of February 2020, several confirmed cases had been identified; consequently, the Japanese government closed all schools [9]. The number of COVID-19 cases increased considerably by mid-March, and the government declared a state of emergency on 16 April 2020 [8,10]. 

From January 2020 to July 2021, Japan faced four waves of SARS-CoV-2 infections. The first wave was 26 January to 31 May 2020, with 16,582 confirmed cases and 898 deaths; the second wave was June 1 to July 31 2020, with 19,120 confirmed cases and 114 deaths; the third wave was 10 October 2020 to 6 March 2021, with 349,344 confirmed cases and 6612 deaths; and the fourth wave was 6 March to 25 June 2021, with 353,227 confirmed cases and 6395 deaths [11].

At first, Japan appeared vulnerable to the COVID-19 pandemic for several reasons, such as (1) the proximity of Japan to China and the high travel volumes between the two countries; (2) heavy population density and high volumes of commuters in large cities; and (3) a high percentage of older people in the population [12]. However, the Japanese government was able to reduce the number of COVID-19 cases and control the spread of the pandemic [13]. The government developed and implemented a comprehensive COVID-19 response, which included (1) decreasing the number of travelers and returnees from key affected areas; (2) increasing testing and medical capacity; (3) framing the Basic Countermeasure Policy, according to suggestions from an expert committee; (4) providing a stronger legal basis for countermeasure policies; and (5) improving economic recovery [12].

Based on the number of confirmed COVID-19 cases, Japan appears to have been more successful than the US in controlling the pandemic. The main objective of this study was to investigate the pandemic diffusion in the two countries. Herein, a time series analysis of confirmed COVID-19 cases was performed to improve understanding of the spreading dynamics of the pandemic in the two countries.

One way to better understand the spreading dynamics of the pandemic is by generating COVID-19 diffusion graphs and using graph theory metrics to analyze them. In this paper, we proposed a methodology called the pandemic diffusion network dynamics (PDND) approach to build diffusion graphs of the COVID-19 pandemic in the US and Japan. These diffusion graphs were developed by analyzing synchronized fluctuations of COVID-19 time series data among different regions. We then applied a graph theory analysis to better understand the spreading behavior of COVID-19 in the US and Japan. In a previous paper, we showed that graph neural networks—based on synchrony of COVID-19 time series data—can improve the accuracy of predicting COVID-19 dynamics [1]. In this paper, we focused on developing COVID-19 diffusion networks based on synchronized fluctuations and analyzing the networks by means of the graph-theoretical approach.

## 2. Diffusion of Infectious Diseases

The spread of an infectious disease normally follows one of the following patterns: (1) contagious spreading, i.e., moving wave-like from the original center to other centers, (2) hierarchical spreading, i.e., moving progressively from large to small centers, and (3) spreading with both contagious and hierarchical components [14,15]. The locations over which spreading occurs can be treated as a graph containing nodes (regions) with links (diffusion process) between them [14]. The spreading patterns in different locations are frequently observed to fluctuate synchronously [16]. These synchronized fluctuations can be measured by different statistics and often indicate connectivity between locations [17]. 

Various researchers have studied the transmission of diseases by means of graph theory. Reference [18] used US influenza-related mortality data to investigate the between-state progression of the influenza pandemic. The research indicated higher pairwise synchrony between populous states through a correlation analysis between locations. Reference [16] focused on the spatial structure of influenza transmission from June 1918 to April 1919 in England and Wales. The study used statistical methods (lag and correlation analysis) to better understand the spatial and temporal characteristics of the pandemic. Reference [19] investigated the spatiotemporal pattern of dengue hemorrhagic fever incidence by collecting a time series dataset containing 850,000 dengue hemorrhagic fever infections, from 1983 to 1997, in 72 provinces in Thailand. The study used cross-correlation functions to provide metrics of the spatial dependency of temporal correlation among time series. In [20], the researchers attempted to determine how influenza spread in space in one cycle of an epidemic. The researchers investigated the spatiotemporal dynamics of influenza and concluded the importance of diffusion over long distances due to global transportation systems.

The assessment of COVID-19 diffusion in our study was addressed in a state comparison setup. This pointed us to a broader network comparison problem—tackling inexact graph matching. Searching for accurate and effective tools to compare networks pushed the research in many different directions, and led to a wide variety of methods and algorithms. Our study explores if the comparison of two networks and the respective phenomena of COVID-19 diffusion can be quantitatively assessed by means of the pandemic diffusion network dynamics (PDND) approach.

## 3. The Pandemic Network Dynamics Approach 

The PDND approach involves investigating the statistical interdependence between COVID-19 time series data. Correlation and coherence analyses are among the most commonly used methods to examine interdependency [21,22]. In the former, a correlation can be calculated between interconnected geographical entities by using their recorded synchronous time series. In the latter, the correlation concepts are applied in the frequency domain [23]. The result of applying the PDND approach to COVID-19 time series data is a diffusion graph, indicating the spread of the pandemic among regions.

## 4. Methods and Procedures

The following steps were used to develop the diffusion graphs based on the PDND approach:

Step 1. Defining the nodes of the graph. For the US graph, 54 nodes represented US states plus New York City, the District of Columbia, Puerto Rico, and Guam; for the Japan graph, 47 nodes represented prefectures.

Step 2. Collecting the time series COVID-19 datasets. To build two COVID-19 diffusion graphs for the US and Japan, we used the number of daily confirmed cases of COVID-19 from 5 January 2020 to 31 July 2021. For the US, we used daily records for all states plus other territories in the US from the Centers for Disease Control and Prevention [6]. For Japan, we used data for all prefectures from the Japan Ministry of Health, Labour, and Welfare [11].

Step 3. Defining the edges of the network. The edges represent connections between nodes. In the COVID-19 dataset, edges indicated a connection between two locations in terms of synchrony of their COVID-19 dynamics. Network edges can be classified as binary or weighted, and can show the directionality among regions (directed or undirected) [24]. We assumed that all geographical entities were connected with each other.

Step 4. Selecting a method to discover synchronized location. A statistical method, correlation analysis, was used to identify if there was a strong relationship between the COVID-19 time series datasets of different geographical entities. Following numerous other studies [21], we adopted a lag of 0 between the analyzed time series.

**Figure 1:**
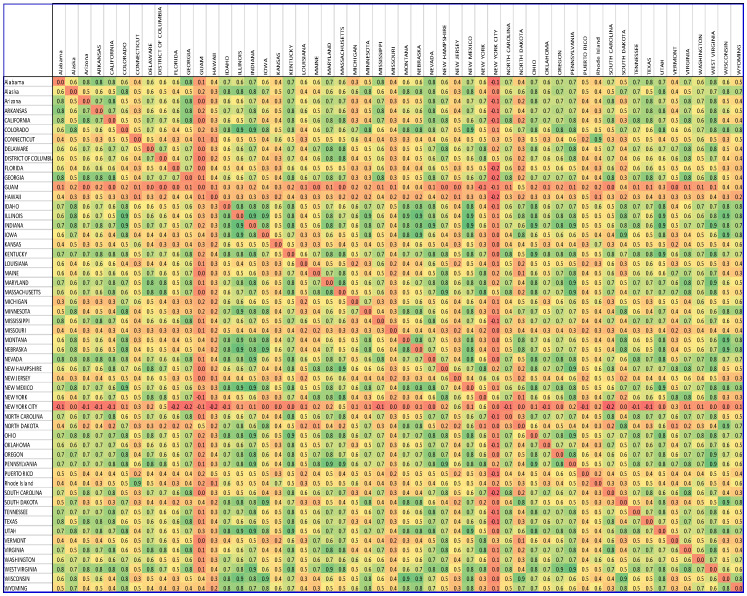
The US COVID-19 adjacency matrix. The green color represents a strong correlation between the time series of the regions, the yellow color represents moderate correlation, and the red color represents a weak correlation. The correlation of each region, with itself, is considered zero.

**Figure 2:**
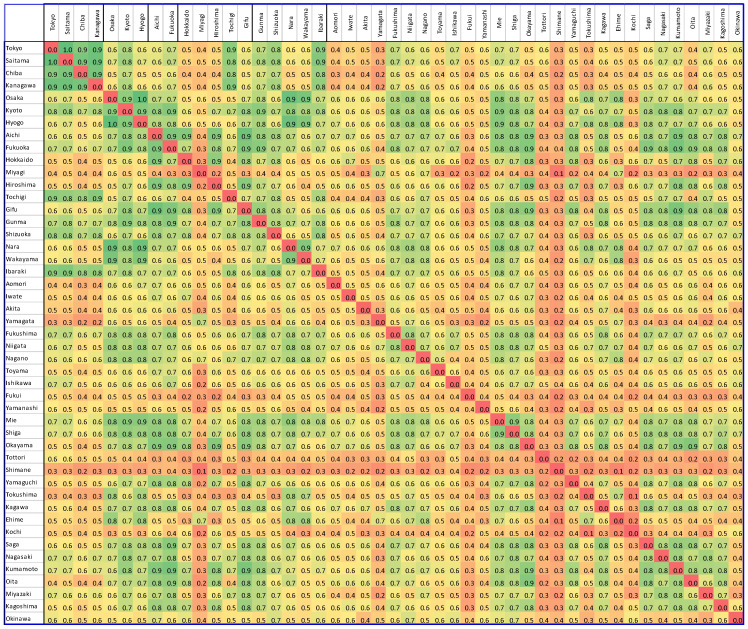
Japan COVID-19 adjacency matrix. The green color represents a strong correlation between the time series of the regions, the yellow color represents moderate correlation, and the red color represents a weak correlation. The correlation of each region, with itself, is considered zero.

Step 5. Forming the connectivity matrix. Computed connectivity between nodes can be used to create a connectivity matrix, which is also known as an adjacency matrix. In this matrix, nodes are represented by rows (i), and columns (j) and edges are represented by matrix entries (aij), as presented in Figure 1 and Figure 2.

**Figure 3:**
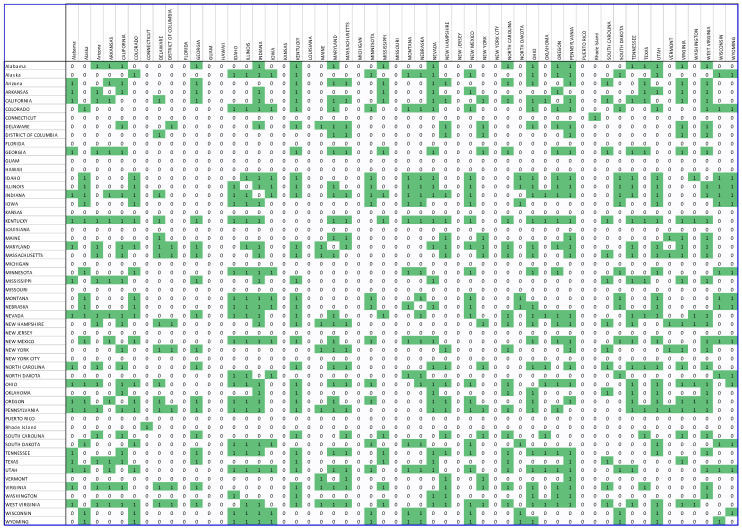
The US COVID-19 binary matrix. The green color represents a strong correlation between the different regions. All strong correlations are represented by 1.

**Figure 4:**
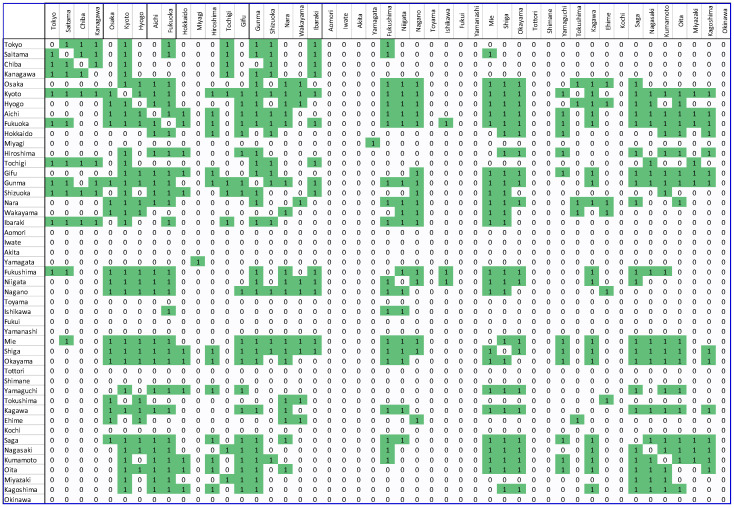
Japan COVID-19 binary matrix. The green color represents a strong correlation between the different regions. All strong correlations are represented by 1.

Step 6. Forming a binary matrix. The adjacency matrix can be used to create an unweighted unidirectional matrix, called the binary matrix. To develop this matrix, a threshold value must be selected. The value of the edge between two nodes was modified to 1 if the value of the correlation between nodes in the connectivity matrix exceeded the threshold, otherwise the value was set to 0. In our study, the threshold value of 0.7 was selected to simplify the network and remove weak and insignificant edges from the matrix. The resulting binary matrices are demonstrated in Figure 3 and Figure 4.

Step 7. Constructing the final diffusion graphs.

Once the PDND graphs are created, it is possible to compute and analyze their topological properties. Global (graph) and local (nodal) graph theory metrics can be used to achieve this. In our study, we selected the clustering coefficient (CC), characteristic path length (PL), local efficiency (Elocal), network density, global efficiency (Eglobal), modularity (Q), and assortativity (r); the nodal measures included degree centrality (K), nodal centrality [24]. First, brief descriptions regarding the adopted network measures are provided in Table 1, along with detailed definitions.

Path length (PL). The average shortest path length was defined as *the average number of steps along the shortest paths for all possible pairs of network nodes* [25]. This metric indicates the efficiency of information transport in a developed network. The average shortest path length of a graph can be calculated with the following equation:

(1) $$l_{G}=\frac{1}{n.\left(n-1\right)}\underset{i\neq j}{\sum }d\left(v_{i},v_{j}\right)$$

In this formula, *d* (*v_i_*, *v_j_*) indicates the length of the shortest path between two nodes. To calculate the average shortest path in a graph, the sum of the shortest paths between all nodes is divided by the number of all possible paths.

Global efficiency (Eglobal). Eglobal, the inverse of PL, is another metric used to quantify COVID-19 spread in a network.

Clustering coefficient (CC). The clustering coefficient is used to better understand the function–structure of the network and is associated with the number of triangles in a network [26]. The clustering coefficient of a graph can be calculated with the following equation:

(2) $$C_{i}=\frac{numberoftrianglesconnectednodei}{numberoftriplescenteredaroundnodei}$$

In this formula, a set of two edges connected to node *i* is called a triple center around node *i*. For the whole graph, the CC is the average of the local values *C_i_*.

Network density. Network density is another metric used to evaluate the effectiveness of a network. This metric is the actual number of connections in the network divided by its maximum capacity [24].

Assortativity (r). Assortativity is used to determine whether high-degree nodes are primarily connected to low-degree nodes or whether nodes with the same magnitude of degree tend to connect to each other [27]. 

Modularity (Q). The modularity metric measures the structure of a network on the basis of the statistical arrangement of nodes [28]. The modularity can have values from −1 to 1, and a value close to zero indicates that the community (modularity) division is not better than that expected at random, whereas a value close to 1 or −1 indicates a strong community structure. The modularity of a graph can be calculated with the following equation:

(3) $$Q=\overset{k}{\underset{i=1}{\sum }}\left(e_{ii}-{a_{i}}^{2}\right)$$

where *e_ii_* is the number of edges that have both ends in community *i*, *k* is the number of communities, and *a_i_* is the number of edges with one end in community *i* [28].

Local efficiency (Elocal). Efficiency in graph theory describes networks from the perspective of information flow [29]. The local efficiency of *a* *graph* is measured as follows:

(4) $$E_{loc}\left(G\right)=\frac{1}{n}\underset{i\in G}{\sum }E_{glob}\left(G_{i}\right)$$

where *E_glob_*(*G_i_*) is the Eglobal of only node *i*’s immediate neighbors, but not node *i* itself [29].

Degree centrality. Nodal centrality quantifies the importance of a node in a network and can be measured by various metrics, such as nodal efficiency, degree centrality, closeness centrality, and betweenness centrality [29]. Among these metrics, degree centrality is one of the most commonly used and is defined by the number of edges of a node. The greater the number of edges, the more central the node.

Apart from the discussed network measures, we also conducted a 0–1 test for chaos to verify the chaotic properties of both US and Japan PDND networks. The test was first proposed by Gottwald et al. [30,31] and later improved by Gottwald et al. [32]. The test results closer to 0 indicate a lack of chaos, while close to 1 indicates the presence of chaotic system properties [32,33]. The whole analysis was carried out in the Python programming language on a single computing machine.

## 5. Results

Most of the metric values computed in the course of the conducted graph theory analysis for both COVID-19 PDND networks are shown in Table 2.

**Figure 5:**
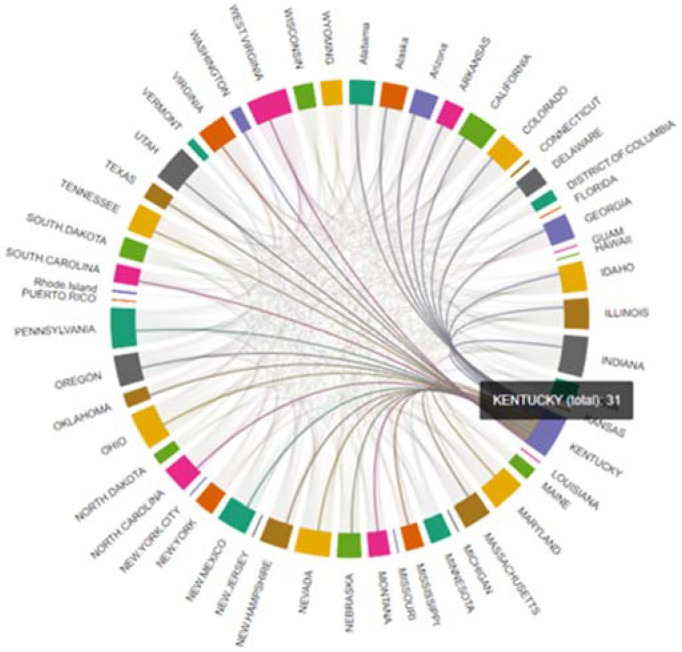
Schematic representation of the COVID-19 pandemic diffusion graph for Kentucky. Nodes (states) are represented in color and the virus-spreading pattern in Kentucky is indicated with lines.

**Figure 6:**
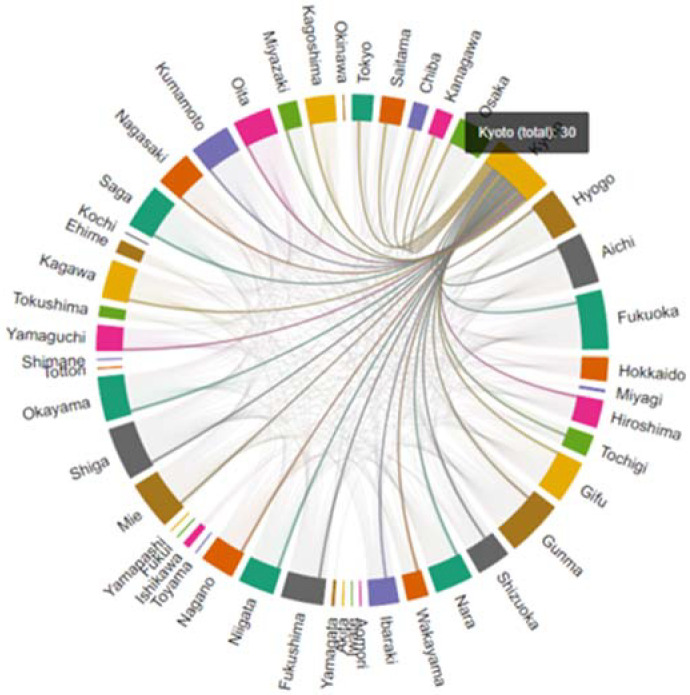
Schematic representation of the COVID-19 pandemic diffusion graph for Kyoto. Nodes (prefectures) are represented in color and the virus-spreading pattern in Kyoto is indicated with lines.

Table 3 and Table 4 and Figure 5 and Figure 6 demonstrate degree centrality for each state and prefecture for the US and Japan, respectively.

## 6. Discussion 

The underlying processes of the pandemic are complex, and understanding them requires analyzing the available COVID-19 data on a global scale. The COVID-19 pandemic diffusion can be considered a nonlinear process that originated in China and spread worldwide [34,35]. Therefore, to identify the main patterns of COVID-19 behaviors, the nonlinearity of COVID-19 data must be taken into account. To discover the insights and implications hidden in COVID-19 data, the applied methods should be adaptive to the underlying nature of the data [35]. To our knowledge, this is the first study to apply synchronized connectivity to analyze the behavior of the COVID-19 pandemic. 

In this study, we developed COVID-19 diffusion networks (graphs) by adopting the PDND approach, and analyzed the graphs properties, including path length, global and local efficiency, clustering coefficient, assortativity, modularity, network density, hubs, and degree centrality.

The path length metric shows the efficiency of information transport in a developed network. A low PL indicates greater integration among geographical regions and the ease of information flow [25]. In the COVID-19 network, the path length represents the diffusion integration of states or prefectures and ease of virus spreading. The average path length for the US COVID-19 network was 1.46, and that for Japan was 1.37. Based on these values, the COVID-19 pandemic spread slightly more easily between prefectures in Japan than between states in the US. A similar observation can be drawn from the global efficiency values of 0.68 for the US and 0.73 for Japan.

The clustering coefficient (CC) is another metric for measuring the ease of information transport in a network, especially on a local scale (states or prefectures) [26]. In general, a higher CC value indicates faster flow of information in the network. As the discussed metric value was 0.72 and 0.74 for the US and Japan, respectively, we conclude that the differences were marginal and do not allow us to draw strong conclusions regarding the speed of virus spread on a local scale.

Similar observations can be drawn based on the computed network density parameter. For the US COVID-19 network, the network density was 0.249, and for Japan 0.253.

Assortativity can be used to determine whether high-degree nodes are primarily connected to low-degree nodes [27]. To calculate assortativity, we used the method described in [30]. The assortativity for the US COVID-19 network was 0.0055, and that for Japan was 0.019. A higher assortativity indicates the preference of a node in a network to connect to others that are similar. Based on the obtained assortativity values, and thorough discussions and demonstrations of networks characterized by various assortativity values available in [36], we conclude that the nature of virus flow in Japan might be slightly more focused on the high-degree hub nodes (prefectures) when compared to the US. However, we note that 0.019 cannot be considered a high assortativity value for the discussed networks.

The modularity metric represents the structure of a network based on the arrangement of the nodes [28]. This metric can have values from −1 to 1, where a value close to 1 or −1 indicates a strong community structure and a value close to 0 indicates a weak and random community structure. The modularity for the US COVID-19 networks was 0.32, and that for Japan was 0.0077. We observe that the analyzed US PDND network was more structured and more module-based than that in Japan. 

The Elocal measures the ability of a network to spread COVID-19 at the local level [29]. A higher Elocal value indicates superior integration and faster transfer of COVID-19 spreading at the local scale. The Elocal for the US COVID-19 networks was 0.83, and that for Japan was 0.84. This outcome does not allow drawing any strong conclusions regarding the analyzed PDND networks.

Degree centrality is defined by the number of edges of a node; the greater the number of edges, the more central the node. In the COVID-19 PDND networks, for Japan, the regional node with the highest degree centrality was Kyoto, and for the US was Kentucky, as represented in Table 3 and Table 4.

Following our previous publication [32], in the case of the (here studied) PDND networks, we evaluated chaotic behavior in the US and Japan using the 0–1 test for chaos. The results were 0.183 and 0.269 for the US and Japan, respectively. From the tangible difference of this metric, we conclude that the spreading of the virus was more chaotic in Japan than in the US. However, in both countries, the absolute value of the test does not allow classifying the pandemic behavior as chaotic.

Based on each adopted measure, it is possible to formulate a general observation that the discovered differences between the analyzed PDND networks were vague and prohibited the formulation of strong conclusions. We believe that such results may be caused by several factors that should be explored in future studies: (1) the proposed graph metrics could not account for subtle differences between networks. In our study, we focused on traditional graph metrics. In contrast, recent progress in the field of graph theory offers a plethora of other metrics and node and graph representation techniques, such as graph node embeddings with Deep Walk [37] or whole graph embeddings [38], as possible examples. (2) The adopted threshold value of 0.7 used for simplification of the adjacency matrix might have caused excessive loss of important information. To verify this hypothesis, a separate search for an optimal threshold value should be carried out. (3) The analysis period was too broad for an approach with a single network representation. The analyzed time series represents almost 18 months and covers several waves of the COVID-19 pandemic. It is possible that virus diffusion patterns evolved over the analyzed time and differed between the waves, for example, in the case of the influenza epidemic studied in [16]. Moreover, as other studies addressing the COVID-19 pandemic distinguished and focused on its various phases [39,40,41], this may indicate that analyzing the whole pandemic in a single procedure may cause bias. If so, the adopted calculation of a single correlation coefficient value between states and prefectures in a too-long period could result in a hindered information extraction process. (4) Influence of lag on the analysis. In our study, a lag of 0 was adopted, as in numerous studies focusing on COVID-19 spread [42,43]. However, it is possible that adopting other lag values will shed more light on the virus spread phenomenon.

## 7. Limitations of the Study

Certain limitations should be acknowledged regarding this study. First, we only focused on two countries: the US and Japan. Investigations of other countries in different parts of the world could produce different results. Second, although we used a correlation analysis to understand connectivity and develop COVID-19 networks, other methods, such as coherence analysis, should also be considered. Finally, the analyzed COVID-19 confirmed case dataset was subject to COVID-19 testing bias, in that the number of confirmed cases was the function of the number of tests conducted in different US states and prefectures in Japan.

## 8. Conclusions

This study adopted the pandemic diffusion network dynamics (PDND) approach to develop COVID-19 diffusion networks for the US and Japan. A graph-theoretical approach was used to understand the behavior of the pandemic in these two countries. The quantitative comparison and assessment of two networks and corresponding COVID-19 diffusion phenomena showed modest benefits of employing the proposed PDND approach. In most cases, the differences between countries measured utilizing adopted graph metrics did not lead to strong conclusions regarding the virus diffusion. Given such findings, we formulated several research hypotheses to be analyzed in future studies, which could determine the utility of the proposed PDND approach regarding the COVID-19 pandemic spread. We hope that other researchers will follow our direction and participate in the opportunity of examining the described methodology.

## Figures and Tables

**Table 1:** Network measures.

Metrics	Description
Path length (PL)	Average of the shortest path lengths over all nodes
Clustering coefficient (CC)	Existing edges/all possible connected edges
Global efficiency (Eglobal)	The efficiency of information transformation among all pairs of nodes, which is inverse of the average characteristic path lengths between all nodes in the network
Local efficiency (Elocal)	Efficiency of all pairs of nodes
Network density	Density of a network
Assortativity (r)	Tendency of a node to connect to other nodes with similar numbers of edges
Modularity (Q)	Combination of nodes that are more connected to one another than the rest of the network
Degree centrality (K)	Number of edges connected to one node

**Table 2:** Values of graph theory metrics obtained for both analyzed PDND networks.

Metrics	US	Japan
Path Length	1.46	1.37
Clustering coefficient	0.72	0.74
Global efficiency	0.68	0.73
Local efficiency	0.83	0.84
Network density	0.249	0.253
Assortativity	0.0055	0.019
Modularity	0.32	0.0077
0–1 test for chaos	0.183	0.269

**Table 3:** Degree centrality of states in the US.

Node ID	State	Degree Centrality
1	Alabama	18
2	Alaska	18
3	Arizona	17
4	Arkansas	14
5	California	22
6	Colorado	20
7	Connecticut	1
8	Delaware	14
9	District Of Columbia	8
10	Florida	0
11	Georgia	18
12	Guam	0
13	Hawaii	0
14	Idaho	21
15	Illinois	22
16	Indiana	29
17	Iowa	14
18	Kansas	0
19	Kentucky	31
20	Louisiana	0
21	Maine	9
22	Maryland	24
23	Massachusetts	20
24	Michigan	0
25	Minnesota	15
26	Mississippi	13
27	Missouri	0
28	Montana	15
29	Nebraska	17
30	Nevada	25
31	New Hampshire	20
32	New Jersey	0
33	New Mexico	22
34	New York	14
35	New York City	0
36	North Carolina	19
37	North Dakota	8
38	Ohio	27
39	Oklahoma	10
40	Oregon	22
41	Pennsylvania	29
42	Puerto Rico	0
43	Rhode Island	1
44	South Carolina	13
45	South Dakota	14
46	Tennessee	20
47	Texas	12
48	Utah	26
49	Vermont	5
50	Virginia	21
51	Washington	8
52	West Virginia	29
53	Wisconsin	14
54	Wyoming	15

**Table 4:** Degree centrality of prefectures in Japan.

Node ID	Prefectures	Degree Centrality
1	Tokyo	10
2	Saitama	11
3	Chiba	7
4	Kanagawa	8
5	Osaka	17
6	Kyoto	30
7	Hyogo	20
8	Aichi	24
9	Fukuoka	28
10	Hokkaido	11
11	Miyagi	1
12	Hiroshima	13
13	Tochigi	10
14	Gifu	20
15	Gunma	27
16	Shizuoka	16
17	Nara	18
18	Wakayama	10
19	Ibaraki	14
20	Aomori	0
21	Iwate	0
22	Akita	0
23	Yamagata	1
24	Fukushima	20
25	Niigata	17
26	Nagano	16
27	Toyama	0
28	Ishikawa	3
29	Fukui	0
30	Yamanashi	0
31	Mie	23
32	Shiga	25
33	Okayama	21
34	Tottori	0
35	Shimane	0
36	Yamaguchi	12
37	Tokushima	5
38	Kagawa	18
39	Ehime	6
40	Kochi	0
41	Saga	21
42	Nagasaki	17
43	Kumamoto	19
44	Oita	18
45	Miyazaki	9
46	Kagoshima	14
47	Okinawa	0

## Data Availability

Not applicable.
